# Nanoscale Visualization of Electrochemical Activity at Indium Tin Oxide Electrodes

**DOI:** 10.1021/acs.analchem.1c05168

**Published:** 2022-03-07

**Authors:** Oluwasegun J. Wahab, Minkyung Kang, Gabriel N. Meloni, Enrico Daviddi, Patrick R. Unwin

**Affiliations:** † Department of Chemistry, University of Warwick, Coventry CV4 7AL, United Kingdom; ‡ Institute for Frontier Materials Deakin University, Burwood, Victoria 3125, Australia

## Abstract

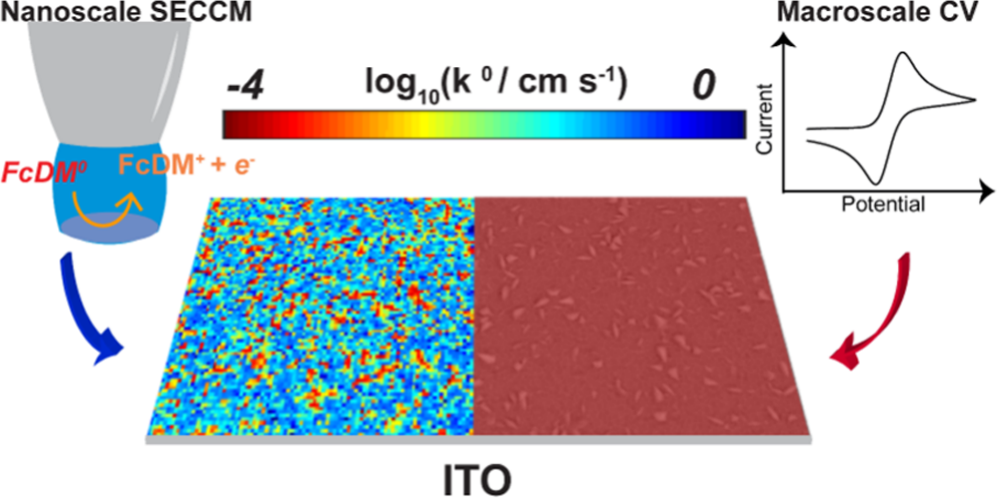

Indium tin oxide (ITO) is a popular electrode choice, with diverse applications in (photo)electrocatalysis, organic photovoltaics, spectroelectrochemistry and sensing, and as a support for cell biology studies. Although ITO surfaces exhibit heterogeneous local electrical conductivity, little is known as to how this translates to electrochemistry at the same scale. This work investigates nanoscale electrochemistry at ITO electrodes using high-resolution scanning electrochemical cell microscopy (SECCM). The nominally fast outer-sphere one-electron oxidation of 1,1′-ferrocenedimethanol (FcDM) is used as an electron transfer (ET) kinetic marker to reveal the charge transfer properties of the ITO/electrolyte interface. SECCM measures spatially resolved linear sweep voltammetry at an array of points across the ITO surface, with the topography measured synchronously. Presentation of SECCM data as current maps as a function of potential reveals that, while the entire surface of ITO is electroactive, the ET activity is highly spatially heterogeneous. Kinetic parameters (standard rate constant, *k*^0^, and transfer coefficient, α) for FcDM^0/+^ are assigned from 7200 measurements at sites across the ITO surface using finite element method modeling. Differences of 3 orders of magnitude in *k*^0^ are revealed, and the average *k*^0^ is about 20 times larger than that measured at the macroscale. This is attributed to macroscale ET being largely limited by lateral conductivity of the ITO electrode under electrochemical operation, rather than ET kinetics at the ITO/electrolyte interface, as measured by SECCM. This study further demonstrates the considerable power of SECCM for direct nanoscale characterization of electrochemical processes at complex electrode surfaces.

## Introduction

Indium tin oxide (ITO) is a versatile optically transparent thin-film conducting oxide with wide applications as an electrode in optoelectronics,^1^ organic photovoltaics,^2^ spectro-electrochemical sensing,^3^ electrocatalysis,^4^ cell biology,^5^ and for super-resolution fluorescence microscopy of electrochemical processes.^6^ These expanding applications are based on the electrical conductivity (about 10^4^ Ω^–1^ cm^–1^) and high transmittance (85%) in the visible region of the electromagnetic spectrum of ITO films, due to the large band gap of about 3.70 eV.^7,8^ ITO films are polycrystalline, comprising grains of nanometric dimensions,^8^ and nanoscale defects.^9^ While ITO is increasingly used as a support for the study of microscopic^3^ and nanostructured entities such as nanoparticles,^10^ nanobubbles,^11^ polymeric nanowire networks,^12^ and carbon nanotubes,^13^ nanoscale electrochemical characterization of ITO surfaces has not been explored.

There is increasing interest as to how heterogeneity in the electrical and electrochemical properties of ITO impacts its performance for the aforementioned applications.^14−16^ While the morphology,^8,15,17^ conductivity,^15,17,18^ spectroscopic behavior,^17,19^ and composition^17,20^ of (modified) ITO surfaces have been characterized down to the nanometer scale, electrochemical measurements have been predominantly performed on the macroscale.^4,21,22^ This “bulk” macroscale electrochemical characterization (usually voltammetry) gives the average activity of the entire electrode surface, although there have been attempts to interpret macroscopic measurements in terms of nanoscale heterogeneous activity, by adopting a partially blocked-electrode model of the surface.^23^ This has led to the description of ITO as having sparsely distributed electrochemically active sites of 50–200 nm dimensions in an otherwise inactive surface.^20,24,25^ The percentage active area deduced from macroscale voltammetry on unetched and unmodified ITO ranges from 0.05 to 1%, which is considerably lower compared to results from conductive-atomic force microscopy (C-AFM) of similarly prepared substrates, where the percentage area of the most conductive sites ranges from 10 to 20%, and the remaining sites have some electrical conductivity.^17,21,26^ Recent scanning electrochemical microscopy (SECM) studies at externally unbiased ITO in the feedback mode, with *ca.* 10 μm spatial resolution (tip size), have revealed variations in electroactivity on a *ca.* 50 μm length scale.^14^

Scanning electrochemical cell microscopy (SECCM) facilitates the direct investigation of electrochemical activity and electron transfer (ET) kinetics at the nanoscale sites of structurally complex and electrochemically heterogenous electrodes.^27^ This scanning probe technique utilizes a mobile meniscus formed at the end of a nanopipette to confine electrochemical measurements to local regions of a substrate. By hopping or scanning the probe across a surface of interest, it is possible to track both electrochemical activity and topography synchronously, thereby allowing the unambiguous visualization of electrochemical processes.^27,28^ This approach has been applied extensively to resolve activity at complex electrodes, including single carbon nanotubes,^29^ individual nanoparticles,^30−33^ composite conductive polymer films,^34^ polycrystalline metal surfaces,^35,36^ highly oriented pyrolytic graphite (HOPG) and graphene,^37^ two-dimensional (2D) materials,^38,39^ polycrystalline boron-doped diamond,^40^ screen-printed carbon electrodes,^41^ and semiconductor electrodes,^42^ among others.

Here, we employ SECCM with a 50 nm diameter nanopipette probe to visualize ET kinetics at ITO substrates of the highest grade (highest conductivity), as commonly used in previous works.^10,11,24^ The SECCM probe size approximates to the grain size in ITO,^8,15^ and thus enables grain-scale analysis of ET kinetics. We study the one-electron oxidation of 1,1′-ferrocenedimethanol (FcDM) as a classical (nominally) fast outer-sphere redox process.^34^ Experiments are complemented with finite element method (FEM) simulations to allow quantitative analysis of experimental data. The results of this study address a knowledge gap in the electrochemistry of ITO at the nanoscale and the relation of nanoscale and macroscale ET characteristics. The understanding gained will be valuable for future use of ITO as an electrode in its own right and as a support in (photo)electrocatalysis, (photo)electrochemistry, and other high-end applications.

## Materials and Methods

### Materials

Potassium chloride (KCl, Honeywell, 99.5%) and 1,1′-ferrocenedimethanol (FcDM, Sigma-Aldrich, 97%) were used as supplied. All solutions were prepared with deionized water (ELGA PURELAB systems; 18.2 MΩ cm at 25 °C). Indium tin oxide (ITO)-coated glass coverslips, 0.17 mm thick, 20 mm × 20 mm, 8–12 Ω/sq resistivity (SPI Supplies, West Chester, PA), were cleaned following typical protocols of sonicating in isopropanol followed by deionized water and then dried in an argon stream.^20,24^

### Nanopipettes, Electrolytes, and Quasi-Reference Counter Electrodes (QRCEs)

Nanopipettes were fabricated from quartz capillary tubes (QTF100-50-10, Sutter Instrument) with dimensions: 1.0 OD × 0.5 ID × 100 L mm. They were pulled to a fine aperture with a CO_2_-laser puller (Sutter Instrument P-2000; pulling parameters: line 1 with HEAT 750, FIL 4, VEL 30, DEL 150, and PUL 80; line 2 with HEAT 650, FIL 3, VEL 40, DEL 135, and PUL 150). The nanopipettes possessed an opening diameter of ∼50 nm, characterized with field emission scanning electron microscopy (FE-SEM) (GeminiSEM 500 system, Zeiss, Germany); representative SEM images can be found in Figure S1. Each nanopipette was filled with 3 mM FcDM in 50 mM KCl, with a QRCE (AgCl-coated Ag wire) inserted from the back. A small droplet of silicone oil (DC 200, Fluka) was added atop the solution in the nanopipette to minimize electrolyte evaporation from the back opening.^43^ The QRCE potential was stable^44^ and calibrated routinely before and after the SECCM measurements against a commercial leakless Ag/AgCl electrode (3.4 M KCl, ET072, eDAQ, Australia), giving a potential of +75 ± 2 mV. All electrochemical results hereafter are presented versus Ag/AgCl (3.4 M KCl), referred to as Ag/AgCl.

### Scanning Electrochemical Cell Microscopy (SECCM)

A home-built SECCM workstation was used, as previously reported;^34,43,45,46^ full details are given elsewhere.^47^ A single-channel nanopipette was affixed to a *z*-piezoelectric positioner (P-753.3, Physik Instrumente, Germany) and moved to the initial scanning position using an *xy*-micropositioner (M-461-XYZ-M, Newport) controlled with picomotor actuators (8303 Picomotor Actuator, Newport). An optical camera (PL-B776U camera, 4× lens, Pixelink, Rochester, NY) provided a visual guide. The working electrode (WE), which was either an ITO-coated coverslip or a nanocrystalline Au, was mounted on the *xy*-piezoelectric positioner (P-733.2 XY, PI, Germany).

**Figure 1:**
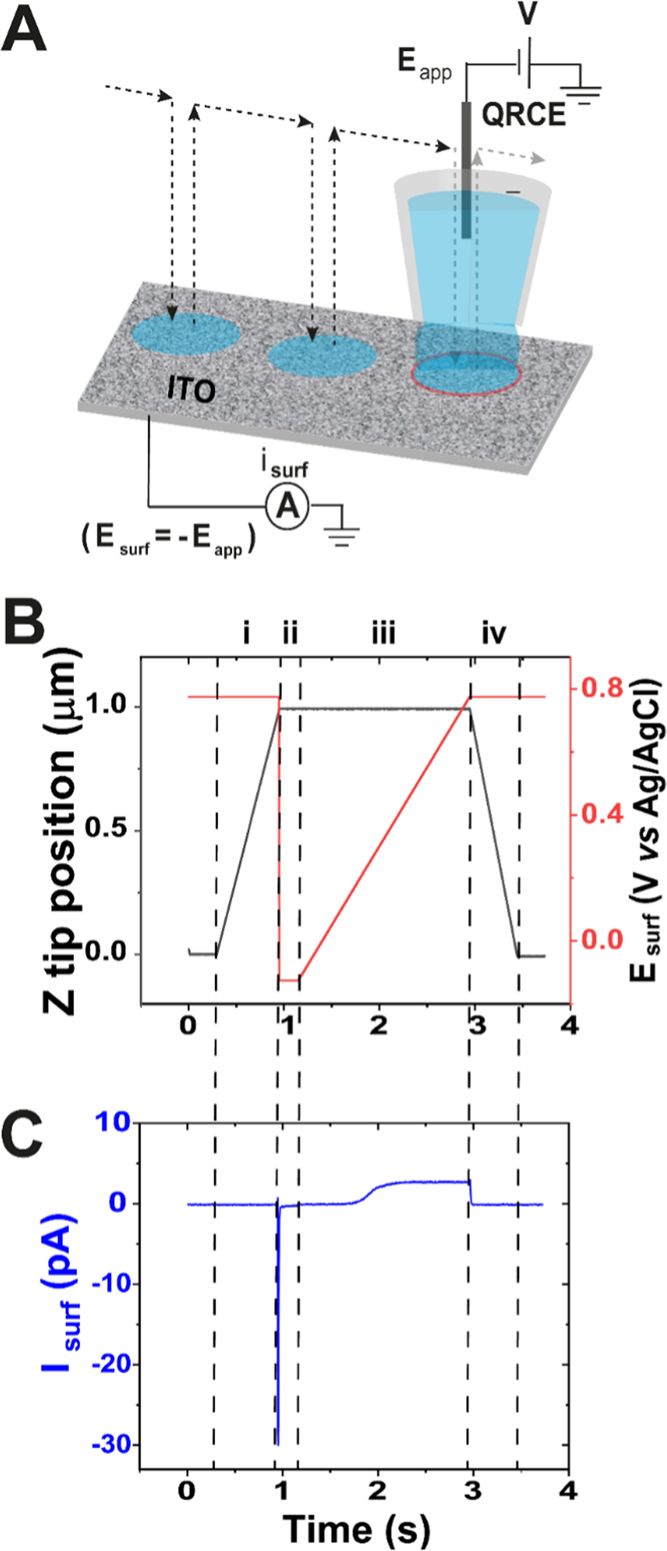
(A) Schematic of hopping mode voltammetric SECCM. A single-channel nanopipette, filled with 3 mM FcDM in 50 mM KCl supporting electrolyte and a QRCE inserted from the back, is translated point-by-point across the ITO working electrode (WE) using piezoelectric positioners (the path of nanopipette is shown as the dotted trace). At each location of meniscus contact, a local voltammetric measurement is made by linearly scanning the potential, *E*_app_, at the QRCE in the probe (equivalent to −*E*_surf_) while recording the surface current (*i*_surf_) at the WE surface. (B) Main features of the imaging procedure during the hop motion of the probe (numbered i to iv) at each pixel. A trace of *z*-position and *E*_surf_ during each step is shown versus time. (C) Current versus time response corresponding to the hop stages in (B). For (B) and (C), the processes are: (i) nanopipette approach toward the substrate surface at *E*_app_ = −0.78 V, to achieve meniscus contact; (ii) switch *E*_surf_ to −0.12 V and hold for 0.2 s; (iii) carry out linear sweep voltammetry at a scan rate of 0.5 V s^–1^; and (iv) nanopipette retraction before moving to the next point. The hop procedure is repeated at the next pixel.

Voltammetric SECCM mapping was carried out with a hopping protocol as illustrated in Figure 1A–C.^27,48^ The nanopipette probe was sequentially approached to the WE substrate at a speed of 1.5 μm s^–1^ [Figure 1B(i)] at a gridded array of predetermined, equally spaced locations. The substrate surface (WE) current (*i*_surf_) measured during this approach stage was zero until the electrolyte droplet at the end of the probe contacted the WE to complete the circuit (*E*_surf_ set to 0.78 V *vs* Ag/AgCl), giving rise to a spike in the *i*_surf_ [Figure 1C(i)], which was used to stop the tip motion (feedback threshold = 0.255 pA). *E*_surf_ switched immediately to −0.12 V and was held at that potential for 200 ms to reset the bulk solution condition [Figure 1B(ii)]. Voltammetric measurements were then executed in the confined area defined by the meniscus cell between the SECCM nanopipette and WE surface, whereby *i*_surf_ was recorded as the potential was swept from −0.12 to 0.78 V at a scan rate, ν = 0.5 V s^–1^ [Figure 1B,C(iii)]. The probe was then retracted [Figure 1B(iv)], and the procedure was repeated at each position, resulting in a spatial- and potential-resolved *i*_surf_ dataset at the WE. The *z*-position of the probe was recorded synchronously throughout, with the value at the end of each approach yielding a topographical map of the WE surface.

Data acquisition and instrumental control were carried out using an FPGA card (PCIe-7852R) controlled by a LabVIEW 2020 (National Instruments, Austin, TX) interface running the Warwick Electrochemical Scanning Probe Microscopy (WEC-SPM, www.warwick.ac.uk/electrochemistry) software. The potential was controlled at the QRCE in the nanopipette (*E*_app_), with respect to ground (e.g., *E*_surf_ = −*E*_app_), and *i*_surf_ at the WE was recorded using a home-built electrometer. Values of *i*_surf_ were measured every 4 μs, and 256 samples were averaged to give a data acquisition rate of 4 × (256 + 1) = 1028 μs (one extra iteration to transfer data to the host computer). All instruments for electrochemical probe positioning and current amplification were placed on a vibration isolator (BM-8, Minus K) and enclosed in an aluminum faraday cage, which was equipped with vacuum-sealed panels (Kevothermal) and aluminum heat sinks to maintain thermal equilibrium during SECCM scans. The faraday cage enclosure was placed on an optical tabletop supported by an active vibration isolation frame (PBI52515, PFA51507, Thorlabs, U.K.).

### Finite Element Model (FEM) Simulations

A two-dimensional (2D) axisymmetric FEM model, representing the geometry of the single-channel nanopipette and the SECCM meniscus, was used to simulate the FcDM^0/+^ redox voltammetry with Butler–Volmer kinetics (see Supporting Information Section S10). From this model, values of the standard rate constant, *k*^0^, and transfer coefficient, α, were deduced from the experimental half-wave potential, *E*_1/2_, and magnitude of the quartile potential difference, Δ*E* = |*E*_3/4_ – *E*_1/4_|, as defined in SI Section S8, at each pixel.

For macroscale voltammetry, DigiElch (v.8.FD, Gamry) was used for simulations in a planar geometry and semi-infinite one-dimensional (1D) diffusion regime. For the ITO substrate, α = 0.5, and *k*^0^ was changed to produce the best fit between the simulated and experimental voltammogram. In all cases, diffusion coefficients of FcDM^+^ and FcDM^0^ were taken as 5.4 × 10^–6^ and 6.7 × 10^–6^ cm^2^ s^–1^, respectively.^49^

## Results and Discussion

### Nanoscale Electrochemical Activity at ITO Electrodes

**Figure 2:**
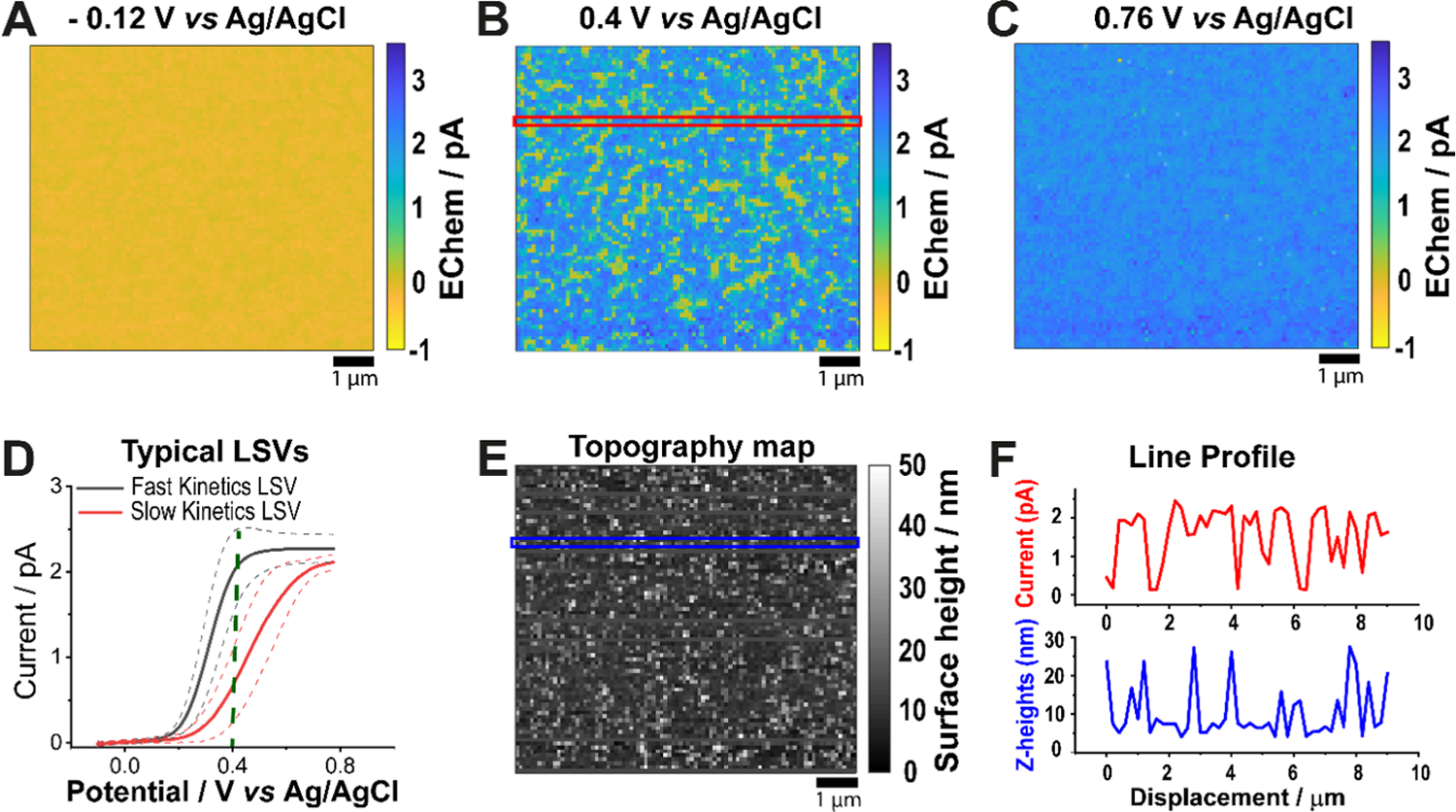
SECCM electrochemical maps (single frames from potentiodynamic movie, SI, Movie S1) of measured voltammetric current at an ITO electrode at *E*_surf_ of (A) −0.12 V, (B) 0.4 V, and (C) 0.76 V. The solution in the nanopipette was 3 mM FcDM with 50 mM KCl. The 9 μm × 8 μm images comprise 7200 pixels, each with an independent LSV collected with SECCM. LSVs selected from different regions on the surface are plotted in (D). Averages of the dominant voltammetric profiles grouped based on the magnitude of Δ*E* = |*E*_3/4_ – *E*_1/4_| (*vide infra*); 6132 LSVs with Δ*E* between 61 and 125 mV were characterized as distinguishable from reversible ET, but having medium to fast kinetics (with the average plotted as a solid black line) and the 1054 LSVs having with Δ*E* > 125 mV were considered to exhibit slower kinetics (with the average plotted as the solid red line). The dashed lines around each average current trace are ±1 standard deviation (SD) of the entire group. The green vertical dashed line at 0.4 V marks the current contrast observed in the electrochemical map in (B). (E) Corresponding topographical map of the ITO surface collected synchronously during SECCM. (F) Plot of the current trace at *V* = 0.4 V (red) and the corresponding *z*-height data points (blue), selected group of pixels covered by the narrow red and blue boxes in (B) and (E), respectively.

Results of an SECCM scan (9 μm × 8 μm area) at an ITO electrode using a 50 nm diameter nanopipette (3 mM FcDM in 50 mM KCl supporting electrolyte) are summarized in Figure 2. At each position (pixel), the FcDM^0/+^ reaction was initiated by a potential sweep from −0.12 V (where no faradaic current flowed) to +0.78 V (well into the diffusion limit) at scan rate ν = 0.5 V s^–1^. The probe hopping distance (*i.e.*, the distance between the centers of adjacent landing sites) was 100 nm. This protocol provided large data sets (1000s of points) from which a series of equipotential electrochemical images of WE current at a set of *xy* coordinates were created. These images were compiled into a potentiodynamic electrochemical activity movie (100 pixels per μm^2^), with 0.51 mV resolution per frame; Supporting Information (SI) Movie S1.

Spatially resolved WE current maps, extracted at potentials, *E*_surf_ = −0.12, 0.4, and 0.76 V, are shown in Figure 2A–C. Evidently, there is significant heterogeneity in electrochemical activity in the kinetic region of the potential scan (0.4 V; Figure 2B). While a fraction of the area has almost attained the diffusion-limited current (*ca.* 2.23 ± 0.22 pA), large patches on the map show currents that are yet to reach 50% of the maximum steady-state diffusion-limited value. These patches correspond to regions of much slower ET and possess a large onset of the half-wave potential (*vide infra*). Conversely, the current measurements in the nonfaradaic region at the foot of the LSV (Figure 2A) and in the diffusion-limited region (Figure 2C) are relatively uniform. It is also important to note that all of the spatially resolved LSVs recorded in the scan presented in Figure 2 (7200 in total) gave a voltammetric response of some kind, indicating that when interrogated directly at the nanoscale, the electrochemical activity of the ITO electrode for a solution redox probe cannot be described as comprising sparse active sites in an otherwise inactive matrix, as has been proposed.^20,24,25^

**Figure 3:**
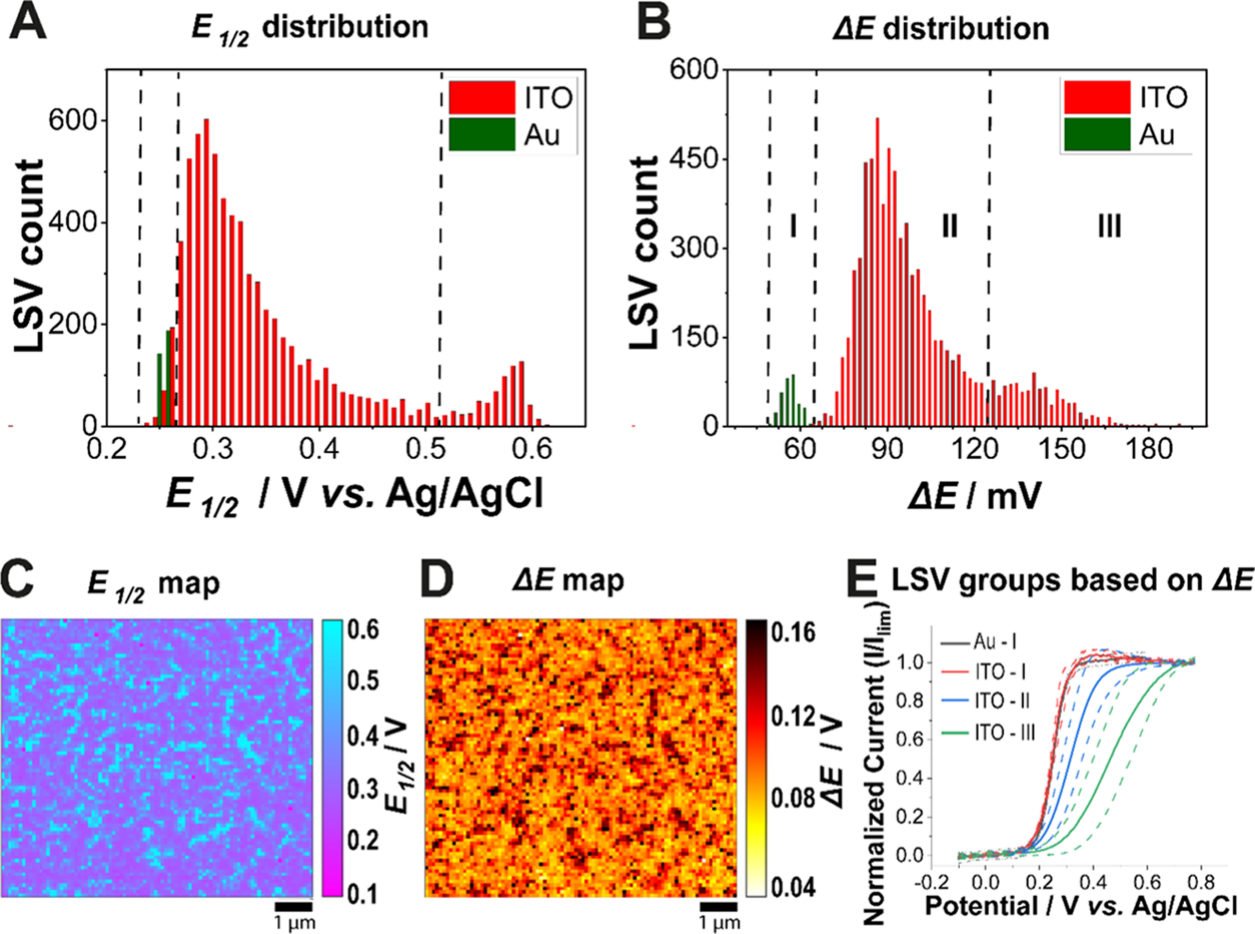
Distribution of (A) *E*_1/2_ and (B) Δ*E* for SECCM LSVs collected on ITO (red) and gold (green) electrodes. Vertical dashed lines on the plots in (A) and (B) section the distribution into the noticeable subpopulations. In (B), such division identifies (I) LSVs on the gold electrode (all showing Δ*E* ≤ 61 mV), (II) LSVs collected on ITO having 61 mV < Δ*E* < 125 mV, and (III) LSVs collected on ITO with Δ*E* ≥ 125 mV. (C, D) Maps of (C) *E*_1/2_, and (D) Δ*E*. (E) Averages of the normalized LSVs according to the grouping in (B) ±1 SD (as dashed lines). The numbers of LSVs averaged were 331 for the gold electrode and 14 for ITO-I, 6132 for ITO-II, and 1054 for ITO-III.

Figure 2D tentatively assigns the SECCM voltammograms to two representative groups, based on the distribution of quartile potential difference, Δ*E* =*E*_3/4_ – *E*_1/4_ (Figure 3B), which was obtained by analyzing individual LSVs. Only a minor proportion of the LSV population (*N* = 14) appears reversible, being comparable to those obtained on Au (*vide infra*), while the remainder exhibit Δ*E* > 61 mV. For convenience, and initial inspection, the LSVs with 61 mV < Δ*E* < 125 mV were grouped as medium to fast kinetics, while voltammograms with Δ*E* > 125 mV were grouped as slower kinetics. For both groups, the FcDM oxidation wave is close to sigmoidal in shape, although with some slight transient effects for the pixels showing the fastest kinetics, before a steady limiting current value is reached. This behavior is also observed in the FEM simulations (see SI Sections S10–S12).^50^ A more detailed kinetic analysis of the SECCM responses is presented in the next section.

SECCM measures the electrochemistry and topography of a substrate synchronously,^27,51^ and the corresponding topography of the ITO scanned area is presented in Figure 2E. The roughness of the SECCM topography map is *ca.* 8 nm RMS in agreement with AFM images of the ITO substrate of the same grade (see SI Figure S2). However, while patterns of ITO crystallites are obvious in the SECCM topography map (and consistent with SEM images in SI Figure S3), it is difficult to ascertain whether there is any correlation between the ITO topography and the heterogeneous distribution of electrochemical activity (Figure 2E). This is further depicted by the absence of any correlative trend in the marginal distribution plot of Δ*E* vs *z*-height data (see SI Figure S6).

Note that the ITO substrate used in this work was not subjected to any surface modification processes, such as oxygen plasma etching and chemical activation with strong acids.^24,52^ Thus, the results presented in Figure 2 are representative of ITO electrodes as would be used practically for electrochemistry. Two additional SECCM scans in other areas of an ITO electrode, emphasizing the reproducibility of the above observations, are presented in SI Figures S4 and S5.

### Statistical Insight into the Spatial Heterogeneity of Electron Transfer Kinetics at ITO versus Au Electrodes

Histograms (Figure 3A,B, red bars) and maps (Figure 3C,D) for *E*_1/2_ and Δ*E* for the scan portrayed in Figure 2 (see SI Movie S1) indicate that although all of the ITO scanned area is electrochemically active, the kinetic distribution is dominated by slower electron transfer (more positive *E*_1/2_ and larger Δ*E*). This is clear from the comparison to a benchmark SECCM scan, at the same spatiotemporal resolution, on a nanocrystalline Au film substrate, with *E*_1/2_ and Δ*E* values extracted in the same way (presented as green bars in the histograms in Figure 3A,B). With *E*_1/2_ = 0.252 ± 0.002 V and Δ*E* = 56 ± 3 mV, as per the Tomeš criterion,^53^ the data for Au indicate complete electrochemical reversibility. SECCM images for the Au scan are presented in SI Figure S8.

For ITO, the subgroups are labeled I, II, and III in the Δ*E* distribution shown in Figure 3B. Of the 7200 ITO LSVs analyzed, only 14 LSVs (*ca.* 0.2%) are apparently (nearly) reversible, showing Δ*E* values similar to those collected on nanocrystalline Au (*i.e.*, Δ*E* ≤ 61 mV, Figure 3E). The prominent category, (II), constituting 85.2% of the total number of LSVs is centered around Δ*E* ≈ 90 mV and *E*_1/2_ ≈ 0.29 V *vs* Ag/AgCl. Subgroup III has a mean Δ*E* of 140 mV and *E*_1/2_ of 0.58 V, making up 14.6% of the population. On the electrochemical maps in Figure 3C,D, regions of “slowest” electrochemical kinetics (*i.e.*, case III) manifest as 50–500 nm sized patterns randomly distributed across the backdrop of case II. Average LSVs (±1 SD), normalized with limiting current (*I*_lim_) at 0.8 V, for all classifications are presented in Figure 3E.

### Estimation of Kinetic Parameters

**Figure 4:**
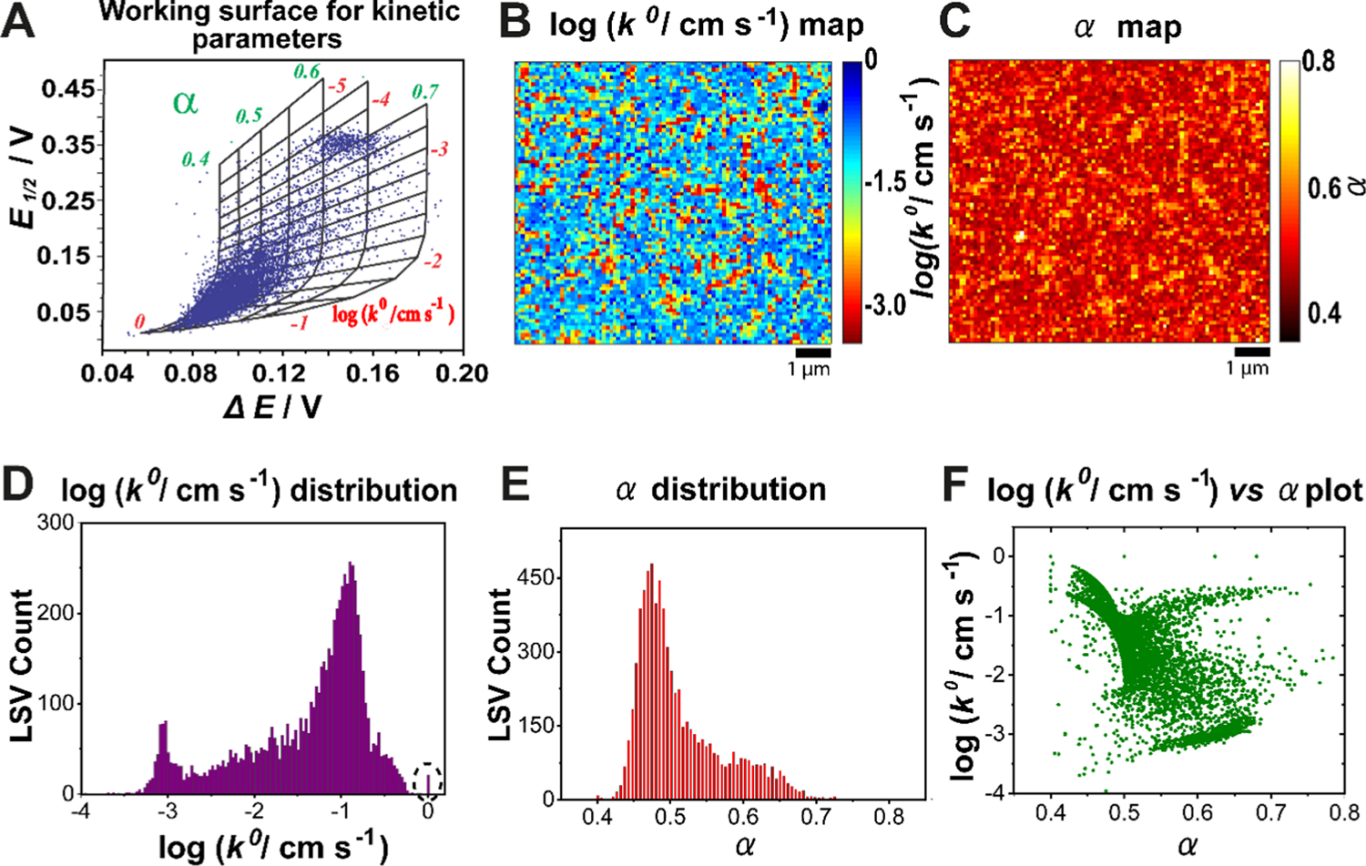
(A) Scatter plot of experimentally derived Δ*E* and *E*_1/2_ overlaid on the kinetic working surface of log(*k*^0^) and α. Maps of (B) log *k*^0^ and (C) α determined from the SECCM scan area (data from Movie S1). (D) Histograms of calculated *k*^0^ values on a logarithmic scale, extracted from (B). A bar representing cases of electrochemical reversibility is circled (dashed) at log(*k*^0^) = 0 (*i.e.*, *k*^0^ = 1 cm s^–1^). (E) Histograms of the corresponding α for the SECCM map in (B). (F) Scatter plot of log *k*^0^*vs* α.

We employed a FEM model^50^ to determine the standard rate constant, *k*^0^, and transfer coefficient, α, at each pixel from the measured *E*_1/2_, and Δ*E*, with formal potential, *E*^0^′, known. A set of 191 LSVs with different combinations of *k*^0^ (in the range of 1 cm s^–1^ to 1 × 10^–5^ cm^–1^) and α (0.4–0.7) were simulated for a nanopipette geometry representative of the one used (details in SI Section S10). Values of Δ*E* and *E*_1/2_ for the simulated LSVs were used to create a working surface (Figure 4A), upon which the experimental data (*E*_1/2_ and Δ*E*) are plotted to give *k*^0^ and α coordinates.^54^

The resulting pixel-resolved log *k*^0^ (Figure 4B) and α maps (Figure 4C) show *k*^0^ values ranging from 1 × 10^–4^ to 1 cm s^–1^, with α in the range of 0.4–0.7. These data are further plotted as a histogram of log_10_(*k*^0^) (Figure 4D). Note that *k*^0^ ≥ 1 cm s^–1^ is experimentally indistinguishable from the reversible case. It is clear from the histogram in Figure 4D that outside the tiny reversible population, there are two main subsets, corresponding to faster (subset II in Figure 3E) and slower (subset III in Figure 3E) ET kinetics. The map and bimodal distribution of α values (Figure 4C,E) which has bimodal centers at α ≈ 0.48 and 0.63 also supports the existence of two different subsets in the estimated α. The range in α is relatively narrowly spread around 0.5, given the large self-exchange electron transfer rate constant for ferrocene and its derivatives.^55,56^ From the scatter plot of log(*k*^0^) and α (Figure 4F), smaller *k*^0^ tends to correlate to larger α, but overall, the picture is complex. It should be noted that this type of method of voltammetric analysis does not necessarily lend itself to accurate determination of α.^54,57^

For the simple FcDM^0/+^ redox probe, the spatial sensitivity of ET kinetics at ITO can reasonably be attributed to variations in the local electronic properties (e.g., local DOS and work function) and nanoscale variations in the nature of the oxide termination of the ITO substrate.^58−60^ From the extracted values of the kinetic parameters, an overall weighted average from the histogram data (7200 individual measurements, bin size 0.0001 and 0.01 for *k*^0^ and α, respectively) of *k*^0^ ≈ 3.61 × 10^–2^ cm s^–1^ and α ≈ 0.53 are obtained for the ITO electrode. The estimates are consistent across other SECCM scans (see SI Figures S11 and S12). To the best of our knowledge, the value of *k*^0^ is the largest reported for a redox process at unmodified ITO and is approximately 2 orders of magnitude larger than for the same redox process measured by macroscopic voltammetry, albeit in acetonitrile solution.^20,24^

We also performed macroscale cyclic voltammetry at an ITO electrode, with 1.1 mM FcDM in the same aqueous electrolyte as used for SECCM. Typical results are presented in SI Section S13 and yield *k*^0^ = 1.5 × 10^–3^ cm s^–1^ (assuming α = 0.5), more than an order of magnitude smaller than the average measured by SECCM. Because SECCM voltammetry draws such a small current (vide supra), it is effectively immune to sample and solution resistance (with sufficient supporting electrolyte) and we can be confident that the kinetic analysis of the intrinsic ET kinetics is free from any other parasitic resistances. Were the ET kinetics measured in SECCM to have translated directly to the macroscale then we would have observed reversible cyclic voltammetry for the range of scan rates presented in Figure S13 in the SI, which is clearly not the case.

A distinction between nanoscale SECCM and macroscale CV is that the former is at the length scale of individual grains in ITO wetted by electrolyte, and the measured working electrode current flows through ITO in the ambient environment to the top contact. In contrast, much of the working electrode current in the macroscopic measurements flows laterally through electrolyte-wetted ITO under bias with the FcDM^0/+^ process occurring, and the conductivity of the electrode will be influenced significantly by the interfacial conditions at the electrode/electrolyte interface.^61^ A recent SECM feedback study of the reduction of FcDM^+^ at unbiased ITO surfaces reveals that the lateral conductivity of ITO is significantly diminished under such conditions,^14^ consistent with our interpretation of the macroscale voltammetric measurements and the slower apparent kinetics to those at the nanoscale.

## Conclusions

Our work has provided an unprecedented view of the nanoscale electrochemical behavior of ITO electrodes. Addressing the ITO surface through a series of 1000s of nanoscale voltammetric measurements for the nominally outer-sphere FcDM^0/+^ ET process has revealed that the entire ITO electrode is active, at a spatial resolution of *ca.* 50 nm, but there are spatial patterns in the ET activity, which we attribute to known nanoscale variations in the electronic properties and the nature of the oxide termination of ITO electrodes. With the aid of FEM models, three major kinetic populations are evident: (i) 0.2% of the ITO surface area exhibits full electrochemical reversibility (*k*^0^ ≥ 1 cm s^–1^, α = 0.5). The majority of the screened ITO sites (85.2%) show slower kinetics (mean *k*^0^ = 4.2 × 10^–2^ cm s^–1^, α = 0.5). Finally, a third group seen as 50–500 nm patches, constituting 14.6% of scanned ITO area, within a higher activity background in electrochemical images, depicts much slower kinetics (mean *k*^0^ = 8 × 10^–4^ cm s^–1^, α = 0.68). The weighted average of these measurements is an electrochemical process with *k*^0^ = 3.61 × 10^–2^ cm s^–1^ and α = 0.53.

Our results clearly demonstrate that ITO is a much more active electrode than previously found based purely on macroscopic measurements. Moreover, the prevailing model of ITO electrodes, as comprising a few sparse active sites in an otherwise inert matrix, does not hold up to scrutiny at the nanoscale. This model was derived from the analysis of macroscopic measurements in terms of a classical blocked-electrode model, but such analysis requires considerable assumptions as to the underpinning model and, consequently, can rarely be unequivocal. In contrast, nanoscale electrochemical imaging provides potentiodynamic movies of spatiotemporal ET activity, from which a wealth of quantitative analyses can be conducted as described in this work.

Comparison of SECCM data and macroscopic cyclic voltammetry measurements in this work has revealed different electrochemical charge transfer resistances operating at different length scales in electrochemical processes. In the case of ITO, our work suggests that kinetic effects at the macroscale are dominated by resistances other than electrochemical charge transfer at the ITO/electrolyte interface, most likely lateral conductivity in the ITO film under electrochemical operation.

## Supporting Information Available

Additional methods (S1); nanopipette characterization (S2); captions for electrochemical Movie (S3); AFM images of ITO (S4); SEM characterization of ITO (S5); additional SECCM scans (S6); correlation of ITO morphology to activity: Δ*E**vs**z*-heights marginal plot (S7); details of quartile potential and Δ*E* estimation (S8); SECCM scan on nanocrystalline Au (S9); details of nanoscale FEM simulation for kinetic analysis (S10); pooled results of FEM kinetic analyses for SECCM scans (S11); comparison of SECCM LSVs with simulated curves (S12); macroscale cyclic voltammetry on ITO (S13); and SI references (S14) (PDF)
Electrochemical flux movie for FcDM^0/+^ process on area 1 of ITO electrode (Movie S1) (AVI)
Electrochemical flux movie for FcDM^0/+^ process on area 2 of ITO electrode (Movie S2) (AVI)
Electrochemical flux movie for FcDM^0/+^ process on area 3 of ITO electrode (Movie S3) (AVI)

## Supplementary Material

ac1c05168_si_001.pdf

ac1c05168_si_002.avi

ac1c05168_si_003.avi

ac1c05168_si_004.avi
