# Supplementary material for: Nanoscale Visualization of Electrochemical Activity at Indium Tin Oxide Electrodes
Source: Anal Chem. 2022 Mar 7;94(11):4729–36. doi: 10.1021/acs.analchem.1c05168 (PMC9007413; doi:10.1021/acs.analchem.1c05168)
Supplement: Supplementary file 1 — ac1c05168_si_001.pdf [file ac1c05168_si_001.pdf]

Supporting Information

Nanoscale Visualization of Electrochemical Activity at Indium Tin Oxide Electrodes

Oluwasegun J. Wahab,[1] Minkyung Kang,[1, 2,*] Gabriel N. Meloni,[1] Enrico Daviddi[1] and Patrick R. Unwin[1,*]

[1] Department of Chemistry, University of Warwick, Coventry CV4 7AL, United Kingdom;

[2]Institute for Frontier Materials, Deakin University, Burwood, VIC 3125, Australia.

** Corresponding authors. Email addresses: m.kang@deakin.edu.au ; p.r.unwin@warwick.ac.uk*

Contents

S1. Additional methods S3

S2. Nanopipet characterization S4

S3. Captions for electrochemical movies S5

S4. AFM images of ITO S5

S5. SEM characterization of ITO S6

S6. Additional SECCM scans of ITO electrodes S7

S7. Correlation of ITO morphology to activity: ΔE *vs.* z-heights marginal plot S9

S8. Details of quartile potential and ΔE estimation S10

S9. SECCM scan on nanocrystalline Au S11

S10. Details of nanoscale FEM simulations for kinetic analysis S12

S11. Pooled results of FEM kinetic analyses for SECCM scans S15

S12. Comparison of SECCM LSVs with simulated curves S17

S13. Macroscale cyclic voltammetry on ITO S18

S14. References S19

S1 Additional methods

*Macroscale cyclic voltammetry***.** Macroscale cyclic voltammetry experiments were performed using a three-electrode cell. The working electrode (WE) was either an ITO substrate or Au film on glass, with a silicone O-ring placed on it to define the geometric area of 0.283 cm^2. Electrical contact to the WE was made externally by means of a copper wire. The O-ring cell was filled with 1.1 mM FcDM + 100 mM KCl electrolyte. Platinum wire was used as the counter electrode while the reference electrode was a commercial leakless Ag/AgCl electrode, as also used to calibrate the SECCM QRCEs. Cyclic voltammetry was performed with a CHI400 potentiostat (CH Instruments Inc., USA).

Scanning electron microscopy. SEM images of nanopipet tips and the ITO substrate were obtained with a Zeiss SUPRA 55-VP FEGSEM and Zeiss SIGMA FE-SEM with In Lens detector. SEM images were collected at 5 keV accelerating voltage.

Atomic Force Microscopy. AFM topography images of ITO coated glass slides were obtained with an Innova AFM (Bruker, USA). An MPP-21100-10 Antimony (n) doped Si probe (Bruker, USA) was used, and the measurements were performed in contact mode. Images was collected with 512 × 512 digital sampling of 100 µm × 100 µm area. Topography maps were analyzed with Scanning Probe Imaging Processing (SPIP) software (version 6.0.14) and different representative regions reported with no interpolation.

S2 Nanopipet characterization

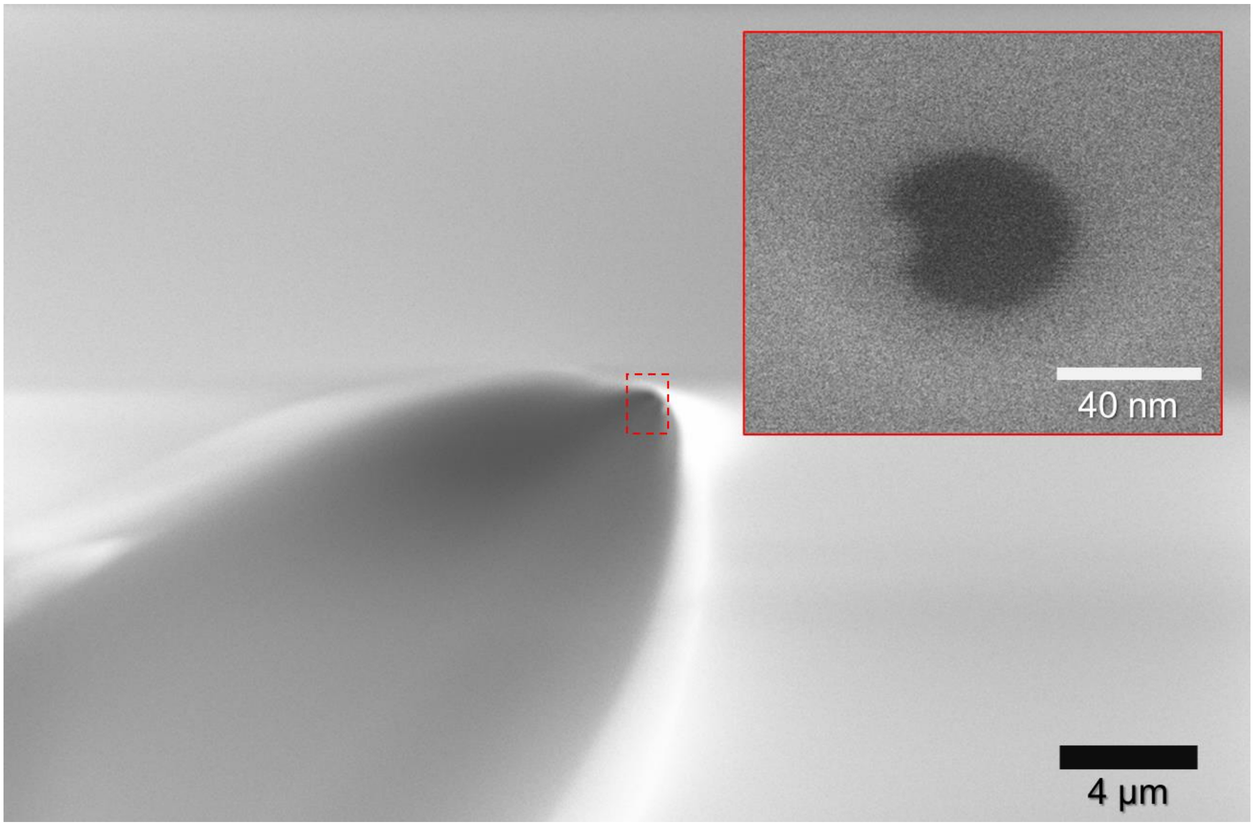

Figure S1. Scanning electron microscopy (SEM) image of representative nanopipet probe (diameter, d ≈ 50 nm) used in the SECCM experiments. The inset shows the top view of the probe's opening.

S3 Captions for electrochemical movies

Movie S1. Electrochemical activity movie of an ITO working electrode (WE) substrate recorded in the SECCM configuration. The movie consists of 90 by 80 pixels (100 nm pitch). Each pixel represents a single linear sweep voltammogram collected by sweeping the potential of ITO from -0.12 V to 0.76 V *vs.* Ag/AgCl at a scan rate of 0.5 V s^{-1}. Each pixel displays the measured WE current at the corresponding potential (movie frame) noted in each frame's title. The electrochemical process visualized is $FcDM^{0/+}$ and the nanopipet was filled with 3 mM FcDM + 50 mM KCl.

Movie S2. As Movie S1, but in a different area of the ITO WE substrate and consisting of 60 by 60 pixels (100 nm pitch).

Movie S3. As movie S1 and S2, but in a different area of the ITO WE substrate and consisting of 50 by 50 pixels (100 nm pitch).

S4 AFM images of ITO

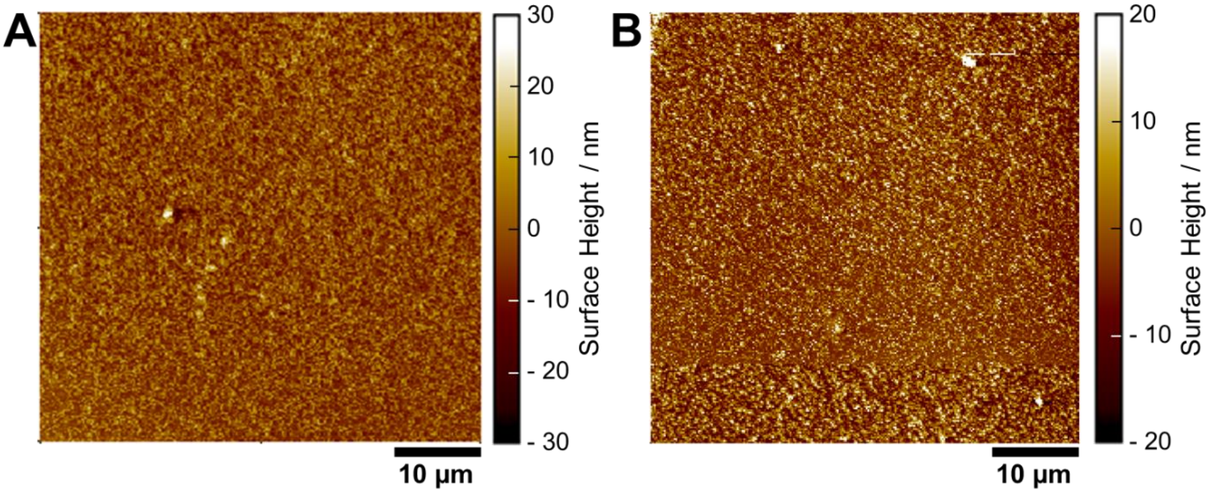

Figure S2. Atomic force microscopy (AFM) topography images of selected areas of the ITO substrate studied with SECCM. Root mean squared roughness = 7 nm ± 2 nm.

S5 SEM Characterization of ITO substrates

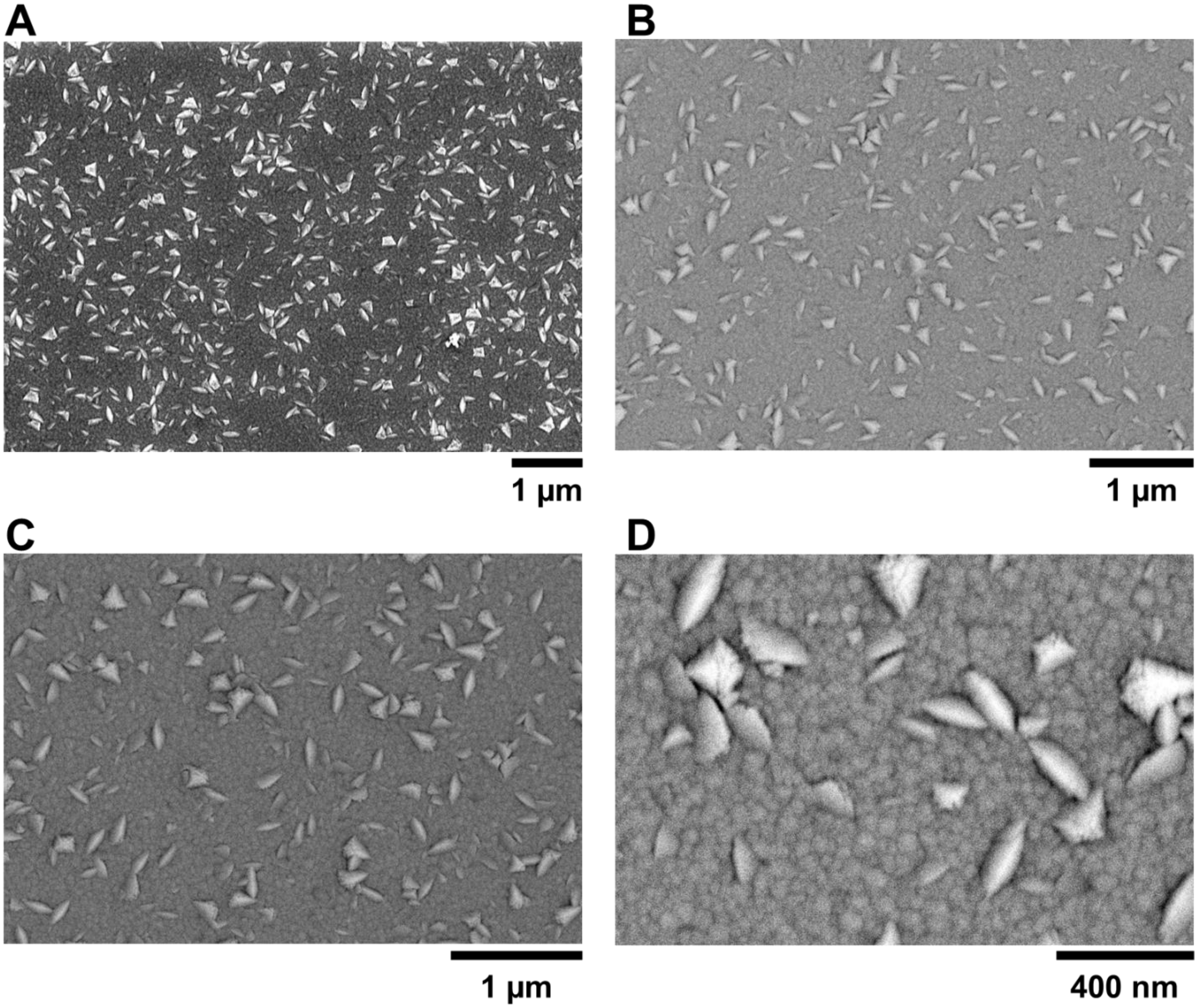

Figure S3. Scanning electron microscopy (SEM) images of ITO surface studied with SECCM. Note the difference in the scale bar from A – D. ITO grains and crystallites can be observed in all the images.

S6 Additional SECCM scans of ITO electrodes

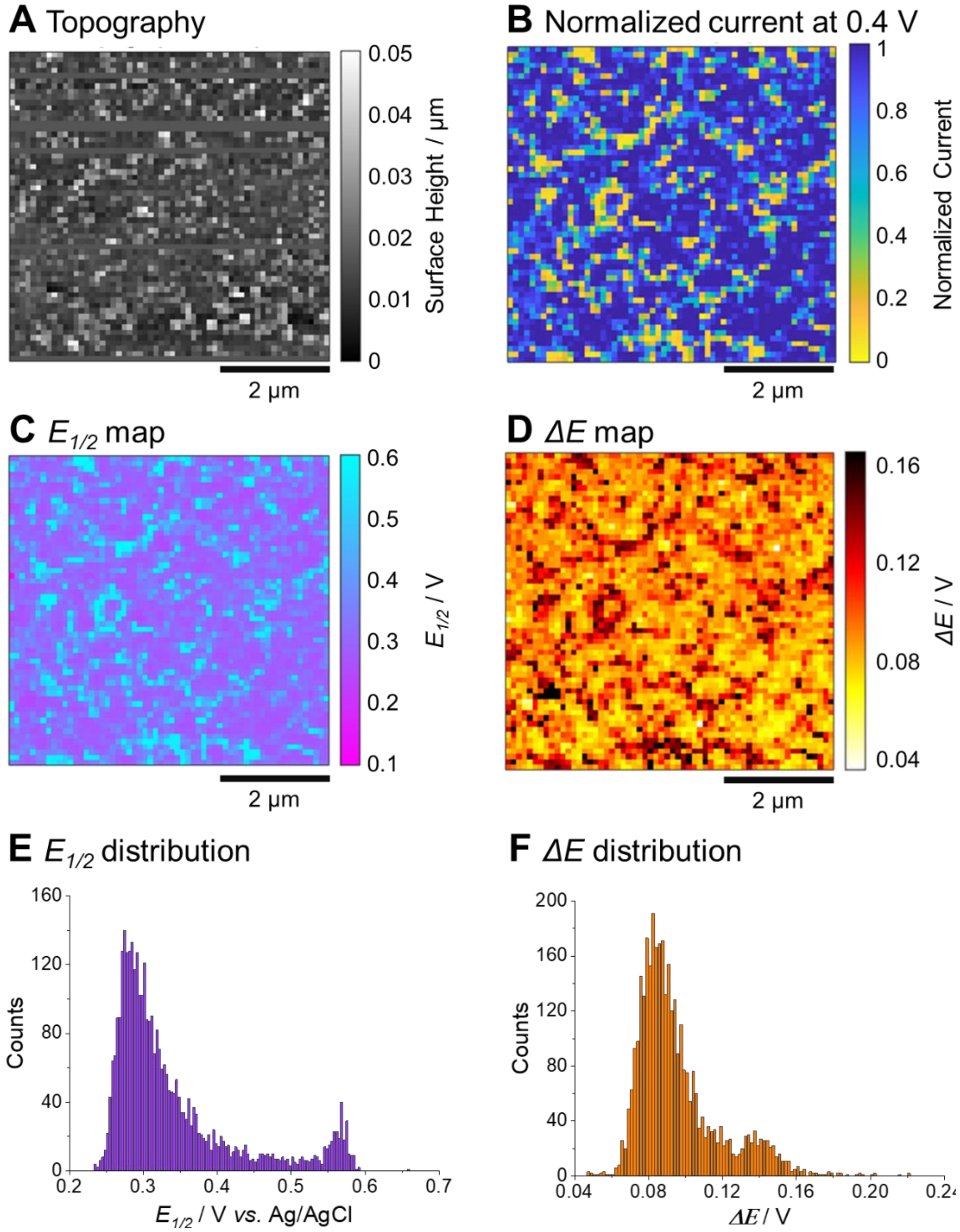

Figure S4. (A) Topography (B) Electrochemical maps at WE potential of 0.4 V *vs.* Ag/AgCl. (C) $E_{1/2}$ map, and (D) $\Delta E = |E_{3/4} - E_{1/4}|$ map acquired in SECCM configuration. Scan is 6 µm by 6 µm recorded with 50 nm diameter nanopipet with hopping distance of 100 nm. Corresponding histogram distributions of $E_{1/2}$ and ΔE are presented in E and F, respectively. Full electrochemical movie showing resolved activity on the ITO WE surface throughout the potential sweep from –0.12 to 0.76 V *vs.* Ag/AgCl is shown in Movie S2.

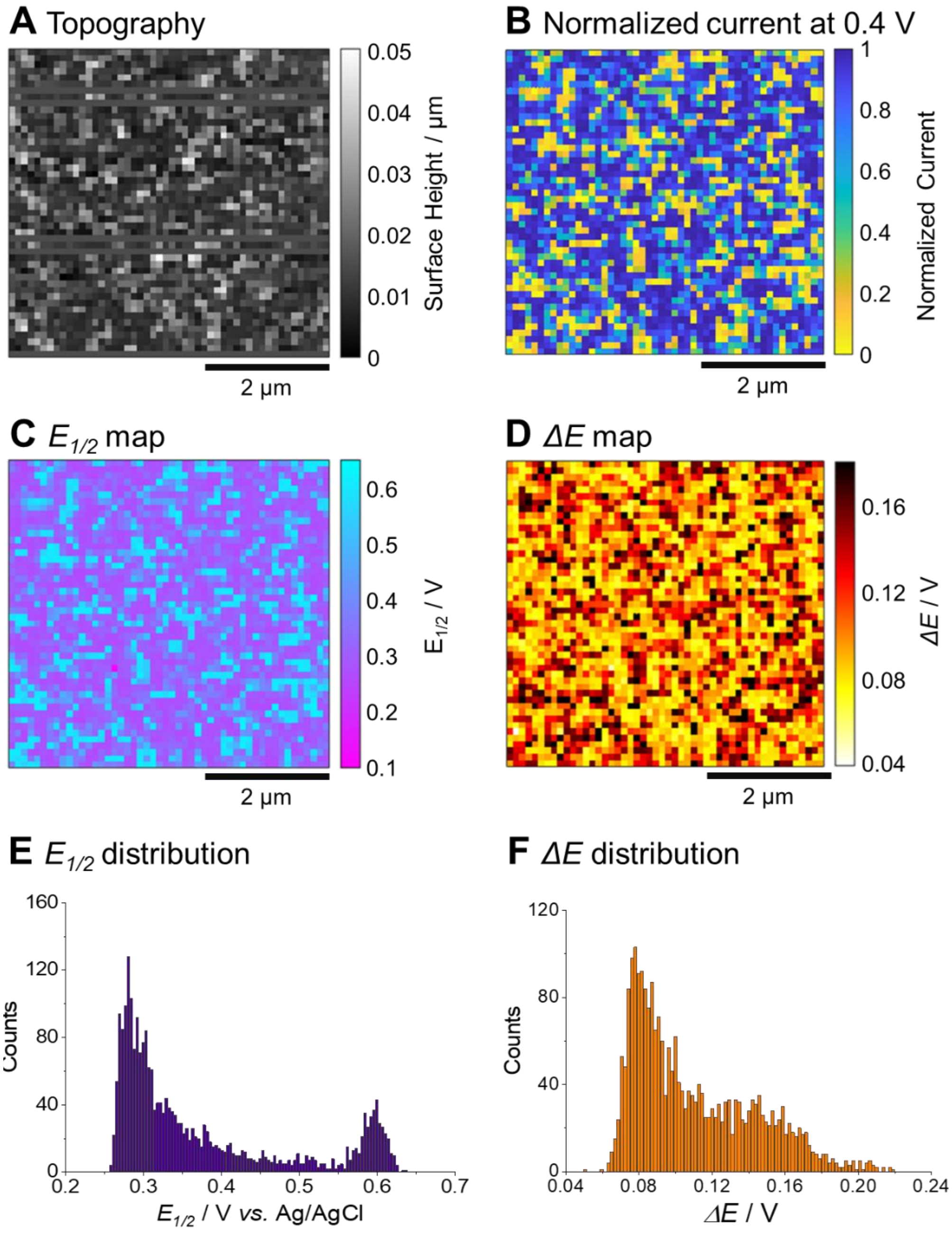

Figure S5. Further results of SECCM scan as in Figure S4, but in a different area of the ITO WE substrate and consists of 50 by 50 pixels (100 nm pitch. Full potentiodynamic electrochemical movie is shown in Movie S3.

S7 Correlation of ITO morphology to activity: *ΔE vs. z*-heights marginal plot

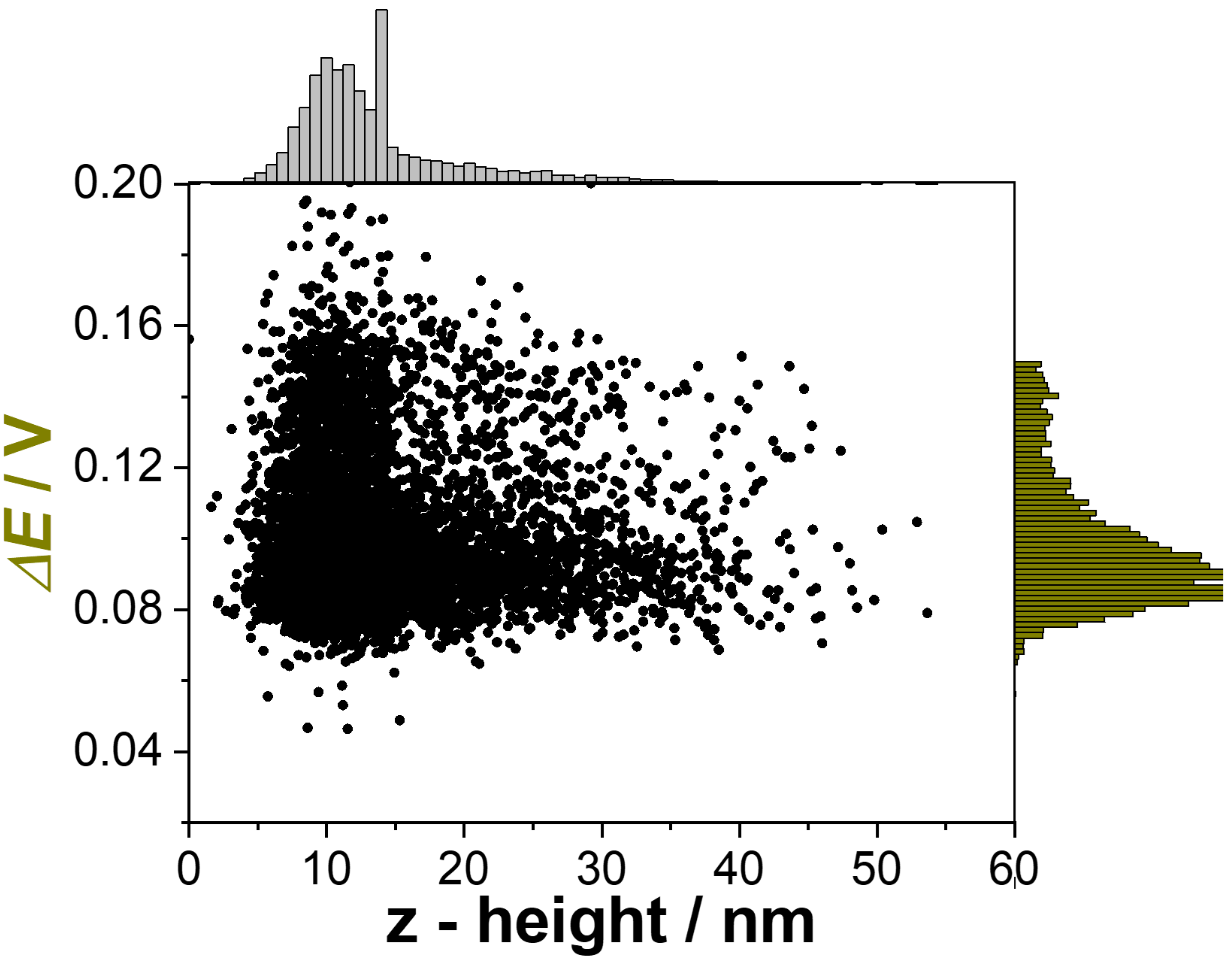

Figure S6. Scatter plots with marginal histogram for *ΔE vs. z* – heights data extracted from SECCM scan discussed in Figure 2 and 3 of main text. Each plotted data point represents the *ΔE* of an LSV recorded with SECCM and the corresponding topographical height (obtained synchronously) of the ITO site.

S8 Details of quartile potential and ΔE estimation

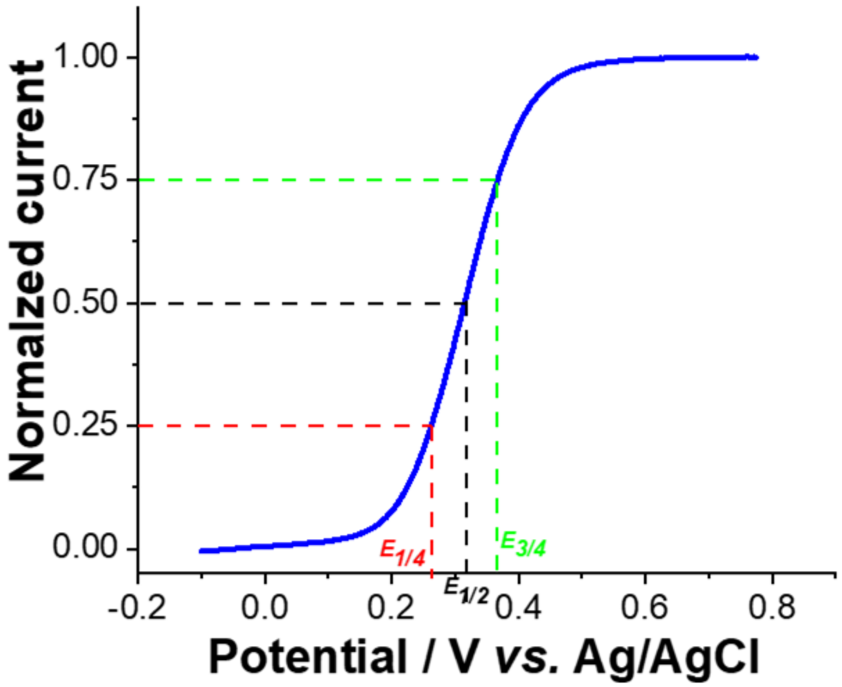

Figure S7. Illustration of the extraction of quartile potentials from an example of normalized steady-state voltammogram measured for $FcDM^{0/+}$ on the ITO WE substrate in the SECCM configuration. The procedure was carried out for each SECCM pixel / LSV to obtain I_{lim}, $E_{1/2}$, ΔE, etc., for kinetic analysis with numerical and FEM simulation approaches.

S9 FcDM Scan on Nanocrystalline Au

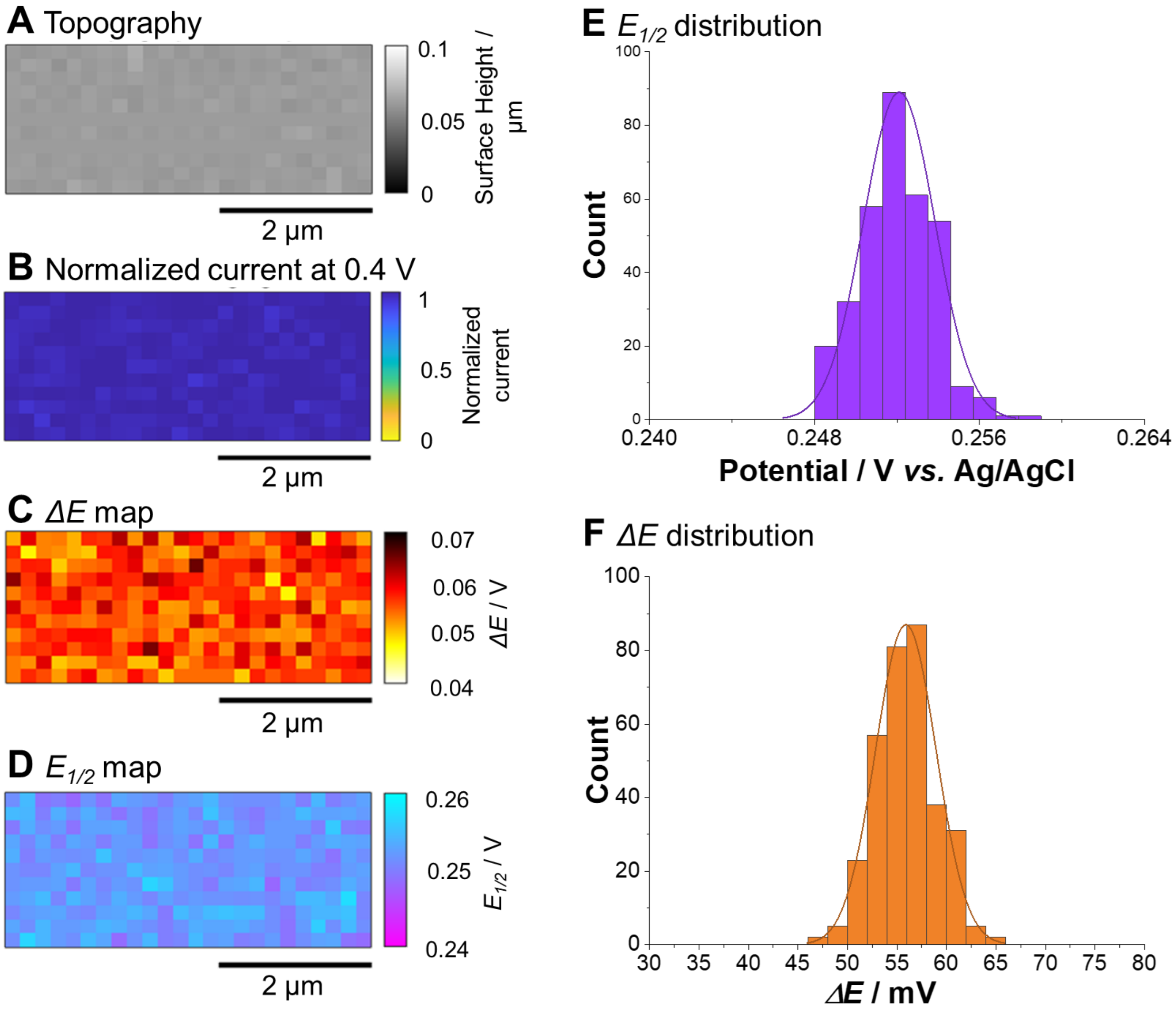

Figure S8. (A) SECCM maps of (A) topography; (B) Normalized current at potential of 0.4 V *vs.* Ag/AgCl (C) ΔE and (D) $E_{1/2}$. Corresponding histogram distribution of (E) $E_{1/2}$ and (F) ΔE. The scan is 6 μm by 6 μm dimension recorded with a 50 nm diameter nanopipet with hopping distance of 200 nm. Probe size, electrolyte composition, and voltammetric scan rate are same as scans on ITO electrode.

S10 Details of nanoscale FEM simulations for kinetic analysis

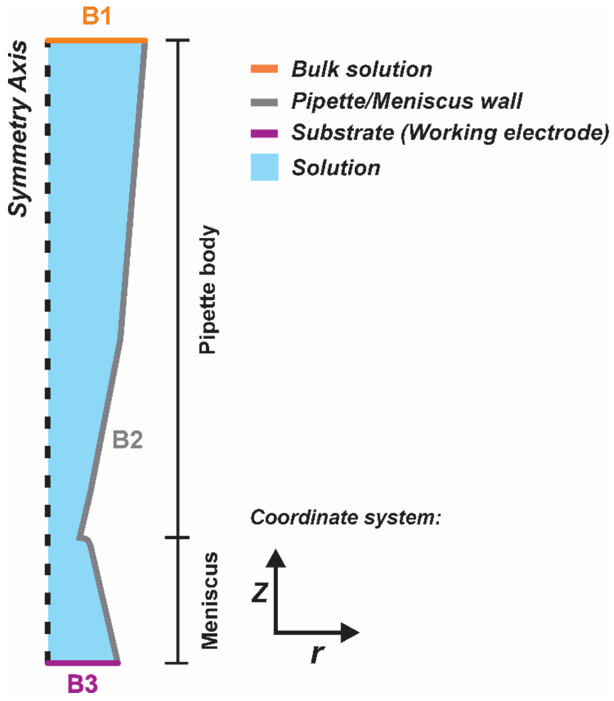

Figure S9. Schematic of the simulation domain used for the FEM model. Boundary conditions are specified in table S1. Drawing not to scale.

Table S1. Summarized boundary conditions for the FEM model.

Boundary	Flux / Concentration condition
B1	$C_{\mathrm{FcDM}^0} = 3$ mM, $C_{\mathrm{FcDM}^+} = 0$ mM
B2	$\boldsymbol{n}\cdot\boldsymbol{J}_{\mathrm{i}} = 0$
B3	$\boldsymbol{n}\cdot\boldsymbol{J}_{\mathrm{FcDM}^0} = (k_{\mathrm{red}}\, C_{\mathrm{FcDM}^+} - k_{\mathrm{oxi}}\, C_{\mathrm{FcDM}^0})$

Table S2. List of parameters used in all simulations.

Symbol	Value	Description
n	1	Number of electrons for $\mathrm{FcDM}^{0/+}$ couple
α	0.4 to 0.7	Transfer coefficient for electrochemical process
k^0	10^{-5} to 1 cm s^{-1}	Standard rate constant
$E^{0\prime}$	0.2298 V	Formal potential of the $\mathrm{FcDM}^{0/+}$ couple
E_{app}	– 0.12 V to 0.78 V	Potential range applied to the WE
v	0.5 V s^{-1}	Potential scan rate
$C^*_{\mathrm{FcDM}^0}$	3 mM	FcDM^0 concentration in bulk solution
$C^*_{\mathrm{FcDM}^+}$	0	FcDM^+ concentration in bulk solution
D_{FcDM^+}	5.4×10^{-6} cm^2 s^{-1}	FcDM^+ diffusion coefficient
D_{FcDM^0}	6.7×10^{-6} cm^2 s^{-1}	FcDM^0 diffusion coefficient

Simulations for the SECCM LSVs were performed in a 2D axis-symmetric geometry, representing the nanopipet (recovered from S-TEM images, see above) and the SECCM meniscus, according to Figure S9. All boundaries within the simulation box are described in Figure S9 and Table S1. Simulation domain size (pipette height = 1.5 mm) and mesh density (> 200.000 elements) were set as such that simulation results were independent of both. The transport of diluted species module was used and mass transport was described by diffusion of the $FcDM^{0/+}$ couple only, assuming no migration due to the high electrolyte concentration to electroactive species ratio used in the experiments (see main manuscript). The potential-dependent flux ($\boldsymbol{J}_{\mathbf{i}}$) for the 1 electron oxidation of $FcDM^{0}$ at the substrate electrode (boundary B3) was described by Butler-Volmer kinetics, where equations S1 and S2 define k_{oxi} and k_{red} employed in Table S2 (boundary B3):

$$k_{\mathrm{oxi}} = k^{0} \exp\left[(1\text{-}\alpha)\frac{F}{RT}\left(E_{\mathrm{app}}(\mathrm{t})\text{-}E^{0\prime}\right)\right] \tag{S1}$$

$$k_{\mathrm{red}} = k^{0} \exp\left[(\text{-}\alpha)\frac{F}{RT}\left(E_{\mathrm{app}}(\mathrm{t})\text{-}E^{0\prime}\right)\right] \tag{S2}$$

A time-dependent solver was used with the driving potential (E_{app}) swept over time using the same voltammetric scan rate (0.5 V s^{-1}) in the experimental framework as was the limits of the potential scan and concentration of species. The formal potential ($E^{0\prime}$) was calculated from the experimental data, using the $E_{1/2}$ value of the 'fastest' LSV in ITO scan (LSV with the smallest $E_{1/2}$ and ΔE), which was identical to Au.

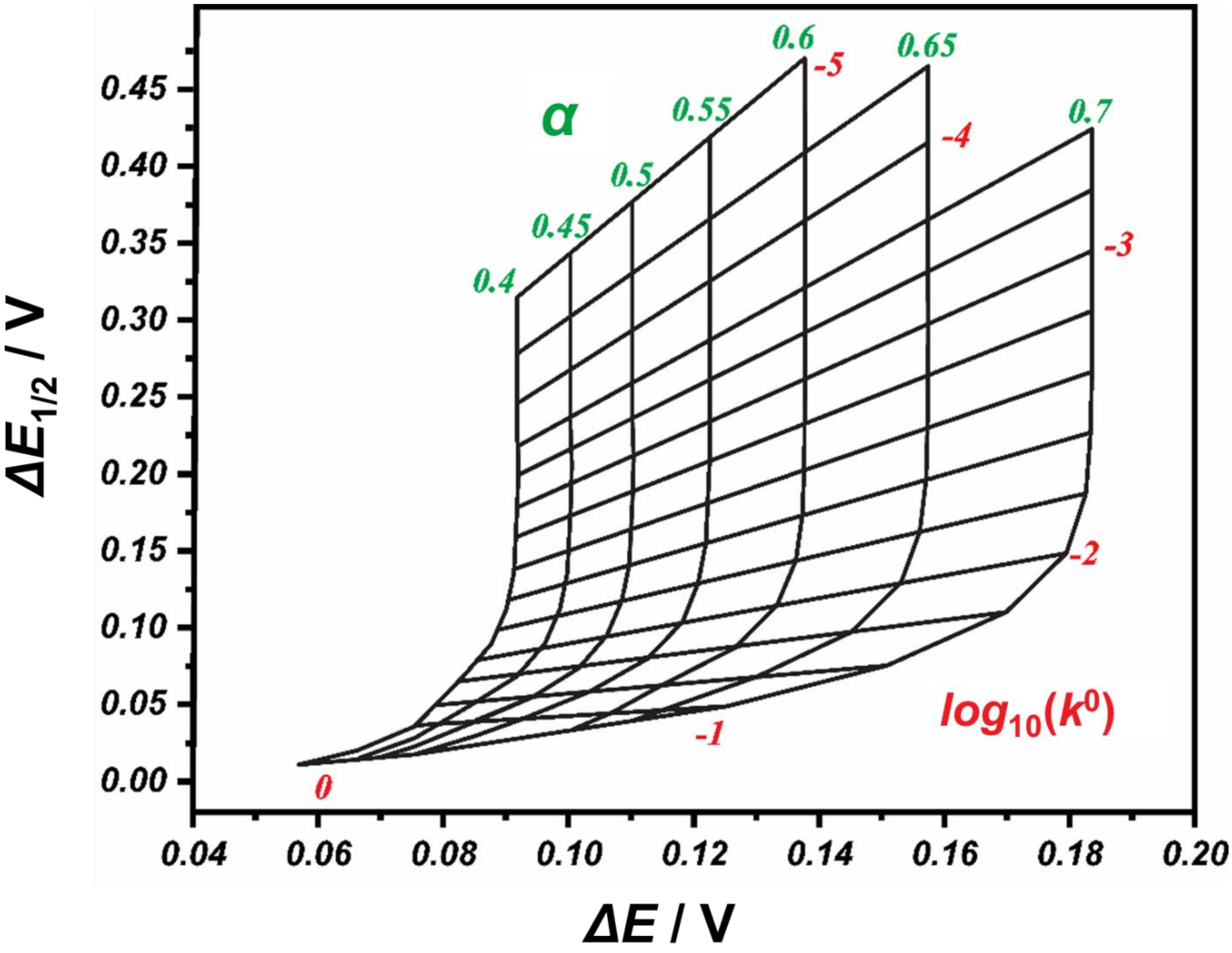

Figure S10. Working surface constructed from the values of $\Delta E_{1/2}$ (corresponding to $E_{1/2} - E^{0\prime}$) and ΔE (corresponding to $E_{3/4} - E_{1/4}$, as defined in Figure S4) extracted from simulated LSVs with different kinetic parameters, k^0 (red numbers, presented as values of log_{10}) and α (green numbers).

A series of 191 simulated *i*-*E* curves for a range of k^0 values from 1 cm s^{-1} to 1×10^{-5} cm s^{-1}, at series of α, ranging from 0.4 to 0.7 were generated from the model; from such curves, values of $\Delta E_{1/2} = E_{1/2} - E^{0\prime}$ and $\Delta E = E_{3/4} - E_{1/4}$ were calculated (see Figure S7) and used to create a working surface[1,2] correlating the kinetic parameters with potential shifts, as seen in Figure S10 (which is presented with reduced data points to facilitate visualization). Extra data points near regions of higher density of experimental points (see Figure 4, main text) were simulated and added to the working surface. A custom script (Wolfram Mathematica v12) was used to interpolate the working

surface and find k^0 and α values from experimental $\Delta E_{1/2}$ and ΔE. This was performed for every single image pixel in the SECCM scans, allowing us to build k^0 and α maps from the experimental, space-resolved LSVs.

S11 Pooled results of FEM kinetic analyses for SECCM Scans

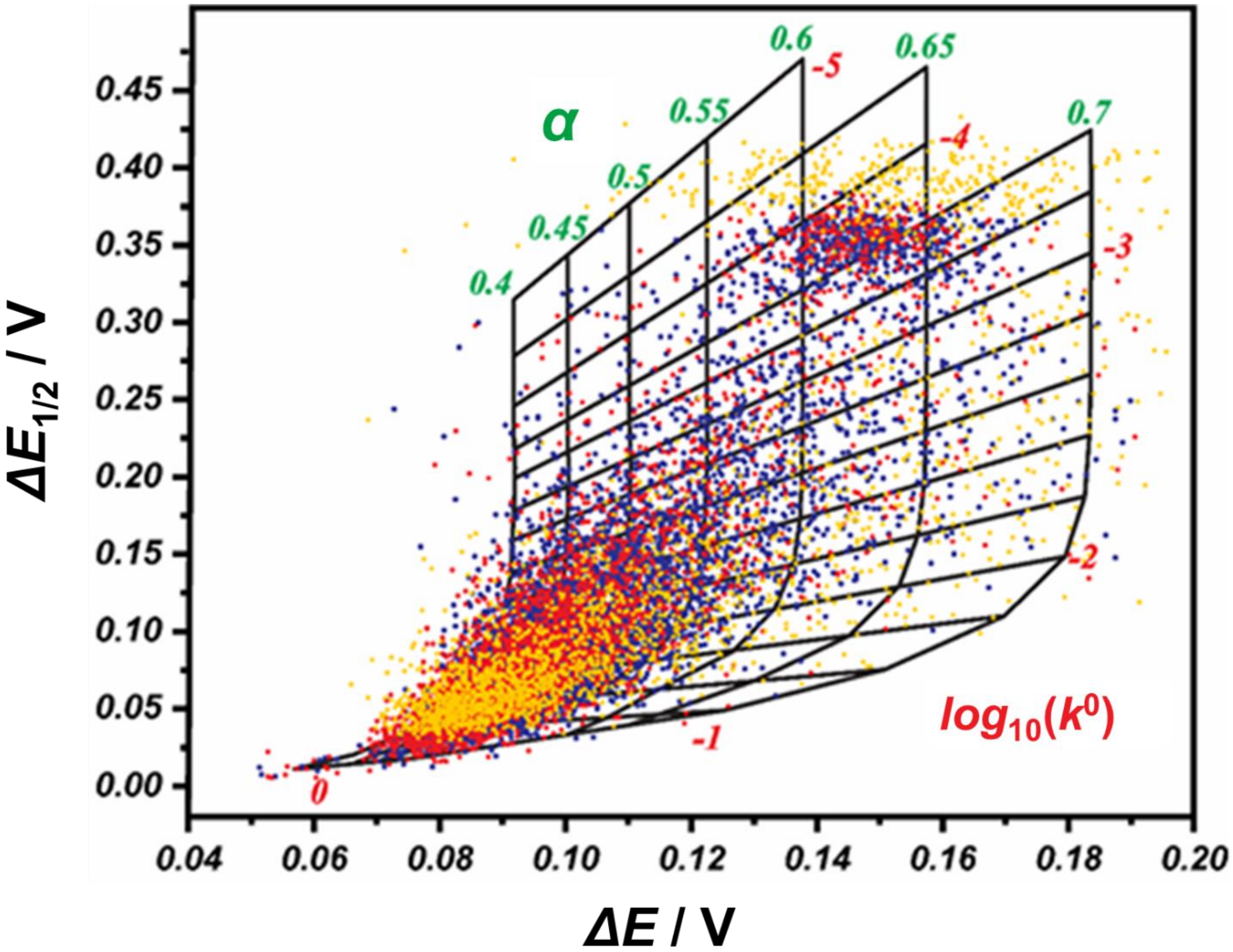

Figure S11. Scatter plots of experimentally derived quartile potentials overlaid on the kinetic indicator working surface simulated for various values of log (k^0) and α. (Figure S10) Data from scans in Figure 3 (main text), Figure S4 and S5 are in blue, red, and yellow respectively. Total independent LSVs analyzed = 13,270.

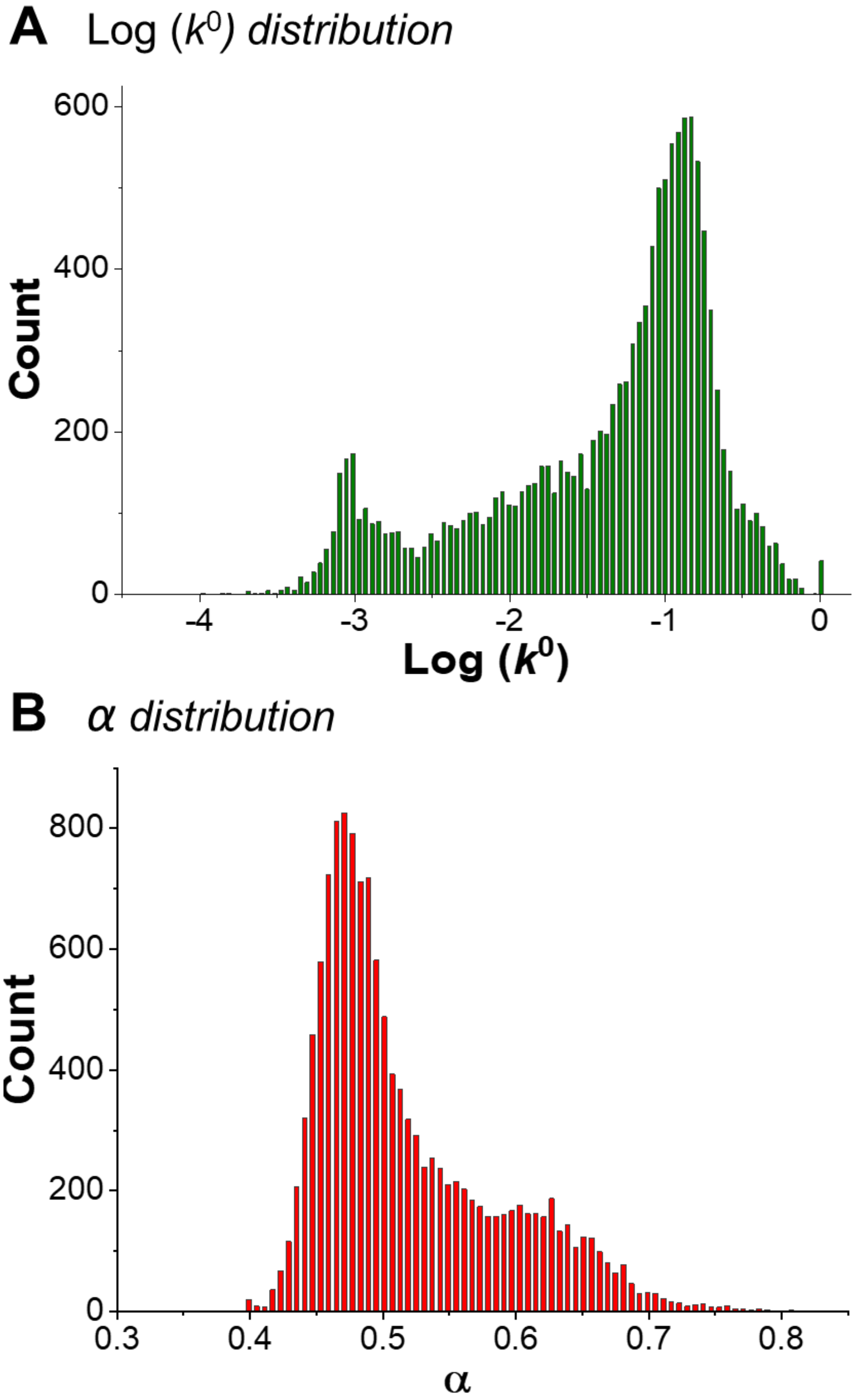

Figure S12. Histogram for (A) k^0 on logarithmic scale and (B) corresponding α for pooled data from three SECCM scans. Total size of dataset = 13,270.

S12 Comparison of SECCM LSVs to simulated curves

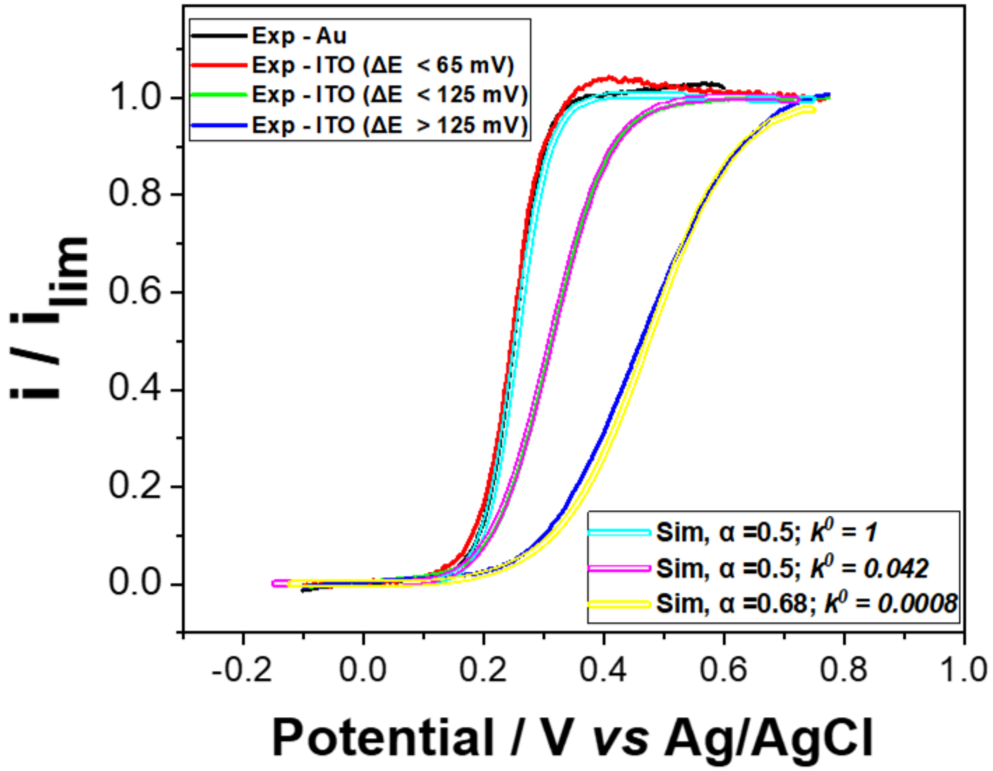

Figure S13. Comparison of normalized current-potential curves that were experimentally acquired with SECCM on Au and ITO substrates to COMSOL simulated $FcDM^{0/+}$ LSVs with different kinetic parameters. The presented SECCM-LSVs for ITO are average of classification by ΔE (see main text). [FcDM] = 3 mM; voltametric scan rate, υ = 0.5 Vs^{-1}; and probe diameter ≈ 50 nm. The fitted kinetic parameters are noted in the legends.

S13 Macroscale cyclic voltammetry on ITO

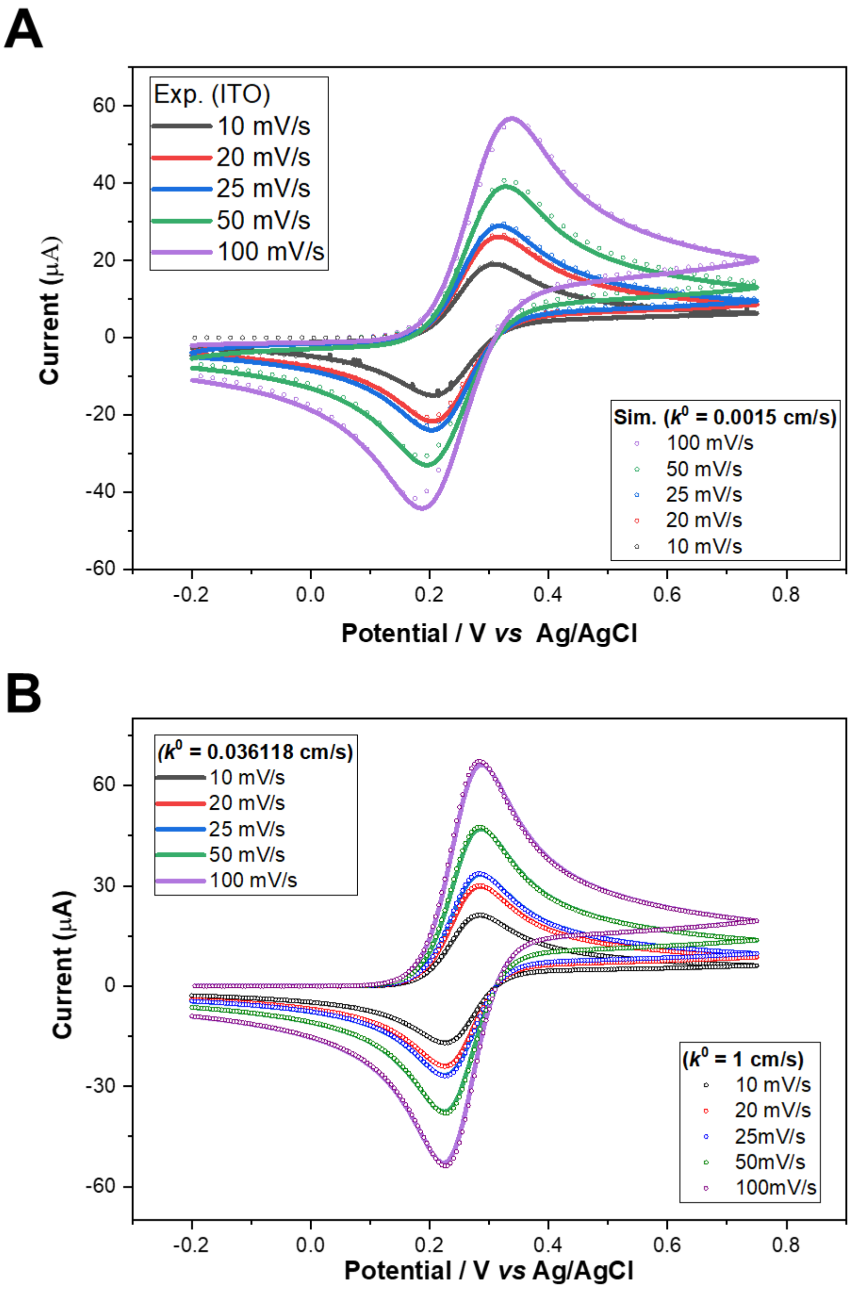

Figure S14. (A) Cyclic voltammograms recorded on ITO in a three-electrode setup at voltametric scan rates of v = 10 to 100 mV s^{-1}. Experimental voltammograms are overlaid with Digi-Elch simulation for α = 0.5, $k^0 = 1.5 \times 10^{-3}$ cm s^{-1}. Electrolyte is 1.1 mM FcDM + 100 mM KCl and electrode area = 0.283 cm^2. **(B)** Simulated macroscale voltammetry comparing weighted average k^0 from SECCM scan ($k^0 = 3.6118 \times 10^{-2}$ cm s^{-1}), k^0 = 1 cm s^{-1} (signifying the case of reversibility). Electrolyte concentration and electrode area used in macroscale experiments were retained.

S14 References

(1) Macpherson, J. V.; Jones, C. E.; Unwin, P. R. Radial Flow Microring Electrode: Investigation of Fast Heterogeneous Electron-Transfer Processes. *J. Phys. Chem. B* **1998**, *102* (49), 9891–9897.

(2) Güell, A. G.; Ebejer, N.; Snowden, M. E.; MacPherson, J. V.; Unwin, P. R. Structural Correlations in Heterogeneous Electron Transfer at Monolayer and Multilayer Graphene Electrodes. *J. Am. Chem. Soc.* **2012**, *134* (17), 7258–7261.
